# RANKL, OPG, and CTS‐K Release in Bone Response to Immediate Nonfunctional Loading of a Single Implant in Mandibular Molar Sites During Osseointegration Establishment

**DOI:** 10.1002/cre2.70193

**Published:** 2025-08-12

**Authors:** Xiaowen Hu, Yijie Fan, Xuexia Chen

**Affiliations:** ^1^ Department of Dental Implant Affiliated Stomatological Hospital of Sun Yat‐sen University, Sun Yat‐sen University Guangzhou China

**Keywords:** cathepsin K (CTS‐K), immediate restoration, osseointegration, receptor activator of nuclear factor‐KB ligand (RANKL), osteoprotegerin (OPG)

## Abstract

**Objectives:**

To verify that osseointegration maturation under immediate restoration is correlative with active release of cytokines.

**Material and Methods:**

The participants needing the restoration of a single missing mandibular molar were randomized into immediate restoration (IR) and conventional restoration group (CR). All eligible patients were recalled for collecting peri‐implant crevicular fluid (PICF) samples according to the scheduled follow‐up time point during osseointegration and functional loading. Detection of receptor activator of nuclear factor‐KB ligand, osteoprotegerin, and cathepsin K in PICF was conducted to statistically analyze their difference between IR and CR groups.

**Results:**

During the osseointegration period, the overall level of these cytokines in the IR group was statistically higher than that of the corresponding cytokine in the CR group. During functional loading, the overall level of the each cytokine in CR was statistically different from that of the corresponding cytokine during osseointegration, but the overall level of each cytokine was not statistically different between the two groups.

**Conclusions:**

The rapid osseointegration maturation under immediate restoration is probably correlative with active release of cytokines related with bone metabolism.

## 1 Introduction

Advancements in biomaterial technology and ongoing clinical research have expanded the options for osseointegration beyond its original prerequisites, leading to a reevaluation of the process as one that now offers reduced treatment times, enhanced esthetics, and increased comfort (Bonato et al. 2022; de Oliveira Fernandes et al. 2022). Immediate loading of a single implant has been extensively discussed as a valid treatment strategy for the posterior regions, with recent innovations in implant designs and surface characteristics (Charifker Ribeiro Martins et al. 2024; Rondone et al. 2024; Gehrke et al. 2021). It boasts a survival rate of 95% to 98.8% in the posterior mandible for short‐term periods (Moraschini and Porto Barboza 2016), and long‐term success has been investigated in both animal (Remísio et al. 2023) and human (Fernandes et al. 2022, 2023; Morena et al. 2024) studies, with encouraging results. Current literature reports no significant differences in implant survival, marginal bone loss, and mechanical or biological complications between immediately and conventionally loaded single implants in the posterior mandible (Mukherjee et al. 2022). With careful patient selection, the immediate loading strategy has gained widespread acceptance, particularly in the healed molar region of the mandible.

Nonfunctional protocols have been incorporated into the immediate loading strategy to safeguard newly inserted implants in partially edentulous patients from exposure to excessive functional or parafunctional forces (Yamagata et al. 2023). Complications such as bruxism and severe clenching increase the risk of failure in the posterior segment of the mandible (Mangano et al. 2017). Studies have reported higher implant survival rates following immediate nonfunctional or delayed loading compared to immediate functional restoration (Kozakiewicz and Wach 2022; Hadzik et al. 2023; Attard and Zarb 2005), especially in the posterior segment of the mandible, where bone of good quality is often present and proper initial implant stability can be readily achieved. However, there is still a concern that immediate loading may only result in a fibrous repair at the interface (Andrade et al. 2022). Therefore, it is imperative to explore the differences in osseointegration between immediate nonfunctional loading and delayed loading in the posterior mandible.

Several histological reports in humans and experimental animals (Lozada et al. 2000; Hong et al. 2022; Yorioka et al. 2024) have indicated the presence of mineralized tissues at the interface of immediately loaded implants. Functional loading, as demonstrated in experimental studies, seems to stimulate bone apposition and accelerate implant osseointegration (Kanayama et al. 2024). Mechanical loading enhances osseointegration, increases bone volume and bone mineral density; moreover, it alters the quality of the peri‐implant bone, resulting in a greater number of osteocytes and a different alignment and degree of orientation of the peri‐implant bone collagen fibers (Ferreira Balan et al. 2024; Kuwano et al. 2025). Wolff stated that there is a direct link between mechanical loading and bone formation. Increased stresses act as a stimulus for new bone formation, while reduced stress tends to result in bone loss (Di Stefano et al. 2006). These findings suggest that bone bonding around the implant may require mechanical pressure to stimulate bone remodeling, and a completely unloaded healing period is not necessary (Iezzi, Orlandi, et al. 2009; Iezzi, Pecora, et al. 2009; Tumedei et al. 2020). The cellular and molecular events that link mechanical pressure to bone bonding around the implant are crucial to study during immediate nonfunctional loading and delayed loading.

Osseointegration is defined as the process of establishing a new, robust bond of tissue between living bone and the surface of implants. Throughout this process, osteoblasts, which are active during bone formation, contribute to the remodeling phase of osseointegration (Iezzi, Pecora, et al. 2009; Tumedei et al. 2020). The osteoclast breaks down old or damaged bone, creating space for the osteoblast to deposit new bone. This process ensures bone quality and facilitates the adaptation of new bone at the surface of implants (Iezzi, Pecora, et al. 2009; Tumedei et al. 2020). Appropriate low occlusal force from immediate nonfunctional loading can exert bone‐protective and anabolic effects on the implant‐bone interface, which in turn leads to the release of a large number of cytokines from the peri‐implant bone tissue. Some cytokines associated with bone metabolism govern this process (Weivoda and Bradley 2023; Tater and Diajil 2023; Gheisary et al. 2022; Duarte et al. 2016; Balu et al. 2024; Berglundh et al. 2018; Houchen et al. 2023; Hu et al. 2019), including receptor activator of nuclear factor kappa‐B ligand (RANKL), osteoprotegerin (OPG), and cathepsin K (CTS‐K). These cytokines are generated by diverse types of bone cells and exist in peri‐implant crevicular fluid (PICF) (Cordioli et al. 2000). PICF is responsive to the physiological state of the peri‐implant hard tissue. Therefore, dynamic analysis of RANKL, OPG, and CTS‐K can act as a sensitive method for monitoring the physiological status of osseointegration under immediate functional or conventional loading.

Although experiments in histology and histomorphometry have confirmed that appropriate mechanical stimuli enhance the maturation of osseointegration, bone volume, and bone mineral density in the presence of occlusal loading (Cordioli et al. 2000), there are few human cases that address the biological molecular events associated with the osseointegration of immediate loading of a single implant in humans. In this study, we hypothesized that appropriate mechanical stimuli induce the local release of cytokines related to bone metabolism in various types of osseous cells, which in turn influences the degree of maturation of osseointegration in the presence of reasonable occlusal loading. In this paper, intensive research was conducted to compare the release of three cytokines during the osseointegration stage and post‐prosthetic functional loading between immediately nonfunctionally loaded and conventionally loaded single implants in mandibular molar sites. This study will aid in a better understanding of osseointegration establishment under immediate restoration therapy, making it more predictable and long‐lasting.

## 2 Materials and Methods

This study was designed as a single‐center, prospective, randomized controlled trial, approved by the Ethics Review Board with identification number KQEC‐2021‐45‐02 and conducted in accordance with the principles of the Declaration of Helsinki as revised in 2013. All patients were recruited based on their need for the restoration of a single missing mandibular molar and were treated by the same certified implant dentist to standardize operative protocols and data collection, thereby avoiding inter‐examiner inconsistency. Informed consent was obtained from all participants in the study.

### 2.1 Inclusion and Exclusion Criteria for Patients and Implants

Patients enrolled in the study should be aged between 18 and 65 years, physically capable of undergoing conventional surgical and restorative procedures, and must have healed implantation sites (teeth extracted or lost at least 4 months before implantation). All recruited patients exhibit sufficient bone quality and quantity on radiographic images, with at least 1.5 mm of bone surrounding the neck of the implanted device in the buccolingual direction and adequate bone height to accommodate an implant length of 10 mm above the superior wall of the inferior alveolar nerve canal. A minimum of 8 mm vertical and horizontal space is required for rehabilitation, whether natural teeth or fixed crowns/bridges are used as opposing dentition. Additionally, good oral hygiene is essential, with a full mouth plaque score of less than 25% and a full mouth bleeding score of less than 20% maintained throughout the entire period. The implants used in the study were Zimmer TSV dental implants from (Zimmer Biomet, America), featuring diameters of 4.1 mm and lengths of either 10 or 11.5 mm. Primary stability, ranging from 30 to 45 N. cm, was achieved through insertion torque.

Additionally, the exclusion criteria included the following: conditions requiring long‐term antibiotic or steroid use; renal failure, severe or uncontrolled metabolic bone disorders, or uncontrolled endocrine disorders; alcoholism or drug abuse, HIV infection, smoking > ten cigarettes (or the equivalent in cigars) per day, or chewing tobacco, local inflammation or mucosal diseases; unhealed extraction sites; pregnancy and lactation; severe bruxism/clenching or persistent intraoral infection; neurologic or psychiatric disorders; the need for an angled prosthetic abutment to correct severe deviation of the axial direction of the implant.

### 2.2 Overall Study Design

Patients were randomized into either the experimental group or the control group upon registration at the clinic, based on a randomization list generated by a computer. The participants were allocated randomly to either the immediate restoration group (IR, experimental group) or the conventional restoration group (CR, control group). After a single Zimmer implant was placed in a healed mandibular molar site in each subject, the IR group received a provisional crown without occlusal contact within 2 days and underwent transmucosal osseous healing protocol for 3 months. In contrast, the CR group received a healing abutment immediately and underwent transmucosal unloaded osseous healing for 3 months. Subsequently, the implants that had successfully osseointegrated in both the IR and CR groups were fitted with a definitive crown that provided full occlusal contact, and they continued to be observed for a 6‐month study period.

**Figure 1:**
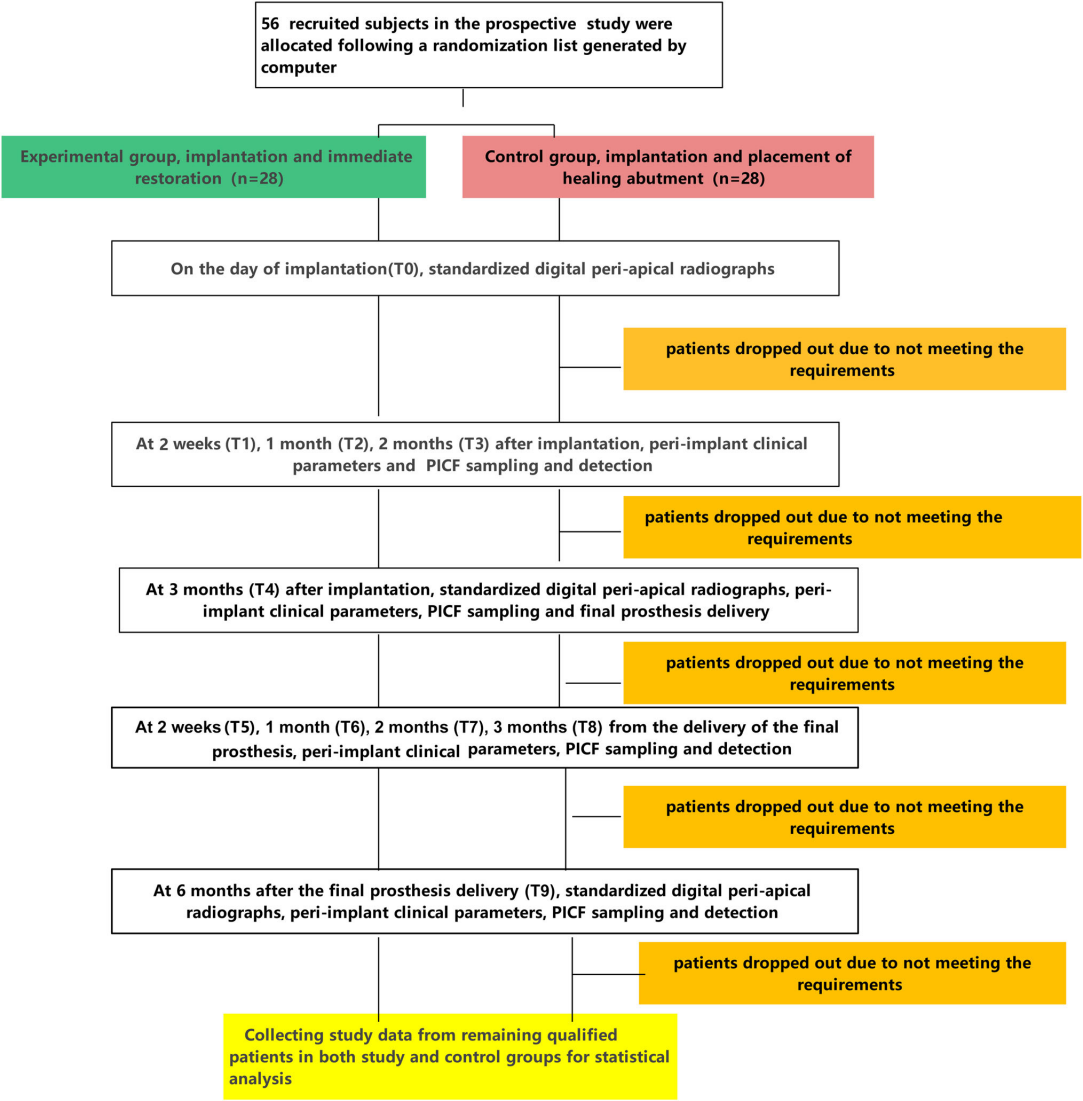
A flow diagram illustrating the activities throughout all phases of the clinical trial design.

During the 9‐month follow‐up period, which included a 3‐month phase of osseous healing (osseointegration) without submersion and a subsequent 6‐month phase of functional loading post‐restoration, all eligible patients were recalled for follow‐up examinations and oral hygiene maintenance visits. These visits aimed to monitor marginal bone loss (MBL) and periodontal health around the evaluated implants, thereby excluding patients who did not meet the inclusion criteria. Therefore, clinical data and PICF samples were collected from the remaining eligible patients at the following intervals: 2 weeks (T1), 1 month (T2), 2 months (T3), and 3 months (T4) post‐implantation surgery, as well as 2 weeks (T5), 1 month (T6), 2 months (T7), 3 months (T8), and 6 months (T9) after the final prosthesis was delivered. The cytokines RANKL, OPG, and CTSK in the PICF collected at these time points were measured in both the immediate loading (IR) and conventional loading (CR) groups. A flow diagram illustrating the activities throughout all phases of the clinical trial design is summarized in Figure 1.

### 2.3 Sample Size Calculation

A power analysis was conducted to ascertain the appropriate number of participants who met the inclusion criteria for enrollment. The effect size was calculated based on the detection limits for OPG and RANKL, as reported in a previous study (Hu et al. 2019). The analysis indicated that a minimum of 20 patients per group was necessary to achieve an error probability *of α* = 0.05 and a power level of 80% (1‐β error probability) in the study. The total sample size was increased by 20% to prevent patient attrition at follow‐up, which could invalidate the study. Consequently, at least 22 patients per group were selected for recruitment into the study.

### 2.4 Surgical Protocol

The surgery was performed under local anesthesia in an outpatient setting, following aseptic protocols. Standard surgical techniques, widely accepted, were used, and the one‐stage surgery protocol was implemented. All implants were placed at the alveolar crest level by the same implant dentist, in accordance with the manufacturer's instructions, while recording insertion torque (IT) values using the surgical motor (Implantmed, W&H, Burmoos, Austria). Patients exhibiting primary stability of their implants at less than 30 N cm or more than 45 N cm at this stage were excluded from further participation in the study. All subjects were required to follow a soft diet to prevent trauma to the surgical sites. Antibiotics were prescribed for 3 days (amoxicillin 1 g twice daily), and ibuprofen 600 mg (200 mg three times daily) as needed for 3 days. Sutures were removed after ten days. Any patients presenting with abnormal peri‐implant status at this stage were also excluded from further participation in the study.

### 2.5 Prosthetic Restoration and Loading Protocol

The provisional crown in the IR group was adjusted for occlusion to ensure it was free from any contact during articulation. Any manipulations other than for occlusal adjustment, retightening loosened screws, or repair were avoided. After 3 months of nonsubmerged osseous healing, only implants that remained stable in the IR and CR groups underwent permanent repair. Definitive cement‐retained ceramo‐metallic crowns were completed within a week, cemented extraorally onto commercially prefabricated straight prosthetic abutments, and then seated intraorally on each successful dental implant, tightened with a torque of 30Ncm. All final prostheses with physiological occlusal contact in both the IR and CR groups continued to undergo a 6‐month follow‐up study. Meanwhile, patients with permanent restorations were instructed to maintain healthy periodontal conditions around the evaluated implants during functional loading. Any subject exhibiting abnormal tissue around the evaluated implant at this stage must be excluded from further study.

### 2.6 Follow‐Up Examinations for Monitoring Peri‐implant Healthy Status

To prevent the negative impact of poor oral hygiene on the cytokines in PICF samples and on peri‐implant MBL, all patients were enrolled in a rigorous follow‐up program until the study's completion. The peri‐implant health index around the evaluated implants was measured at each scheduled time point (from T1 to T9), encompassing the assessment of the following parameters:

- Modified Plaque Index (mPI) on the mesial, distal, buccal, and lingual‐palatal surfaces of the implants (Mombelli and Lang 1994).
- Modified Sulcus Bleeding Index (mSBI) on the mesial, distal, buccal, and lingual‐palatal surfaces of the implants (Mombelli and Lang 1994).
- Probing Depth (PD) (Berglundh et al. 2018), measured to the nearest millimeter with a Hu‐Friedy PGF‐GFS periodontal probe (Hu‐Friedy, Chicago, IL) at the mesial, distal, buccal, and lingual‐palatal sides of the implants.

Patients were attended by a specially trained dentist who recorded the peri‐implant health indices, including the presence or absence of inflammation. At each follow‐up time point throughout the entire study period, the peri‐implant health status was assessed using the mPI, mSBI, and PD, according to their corresponding score criteria (Berglundh et al. 2018; Mombelli and Lang 1994). If peri‐implant mucositis or peri‐implantitis was detected in the implant, it was immediately excluded from the study.

### 2.7 Radiographic Evaluation

**Figure 2:**
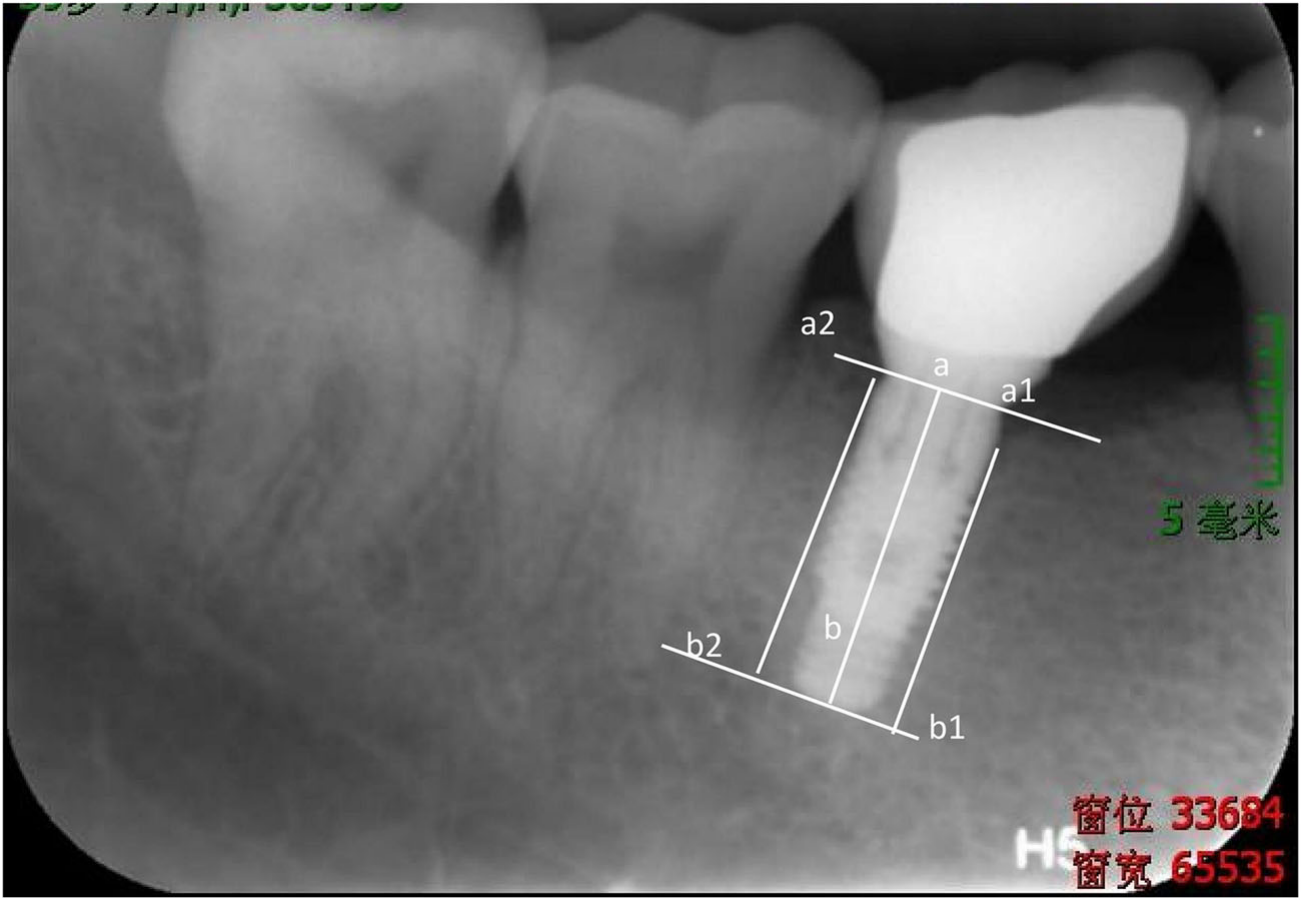
Radiographic measurements on MBL. Point b is the apical point of the end of the implant. Points b1 and b2 are the apical points on the mesial and distal sides of the end of the implant, respectively. Point a is the central point of the implant shoulder platform. Points a1 and a2 are the most apical initial points of the first visible bone contact on the mesial and distal sides of the implant, respectively. MBL at the mesial or distal side of the implant is calculated from the distance between a and b minus the distance between a1 and b1 or between a2 and b2, respectively.

Standardized peri‐apical radiographs were taken at baseline (immediately after surgery), 3 months postimplantation surgery (T4), and 6 months after the permanent restoration was fitted (T9). The radiographs were captured with the film positioned parallel to the implants and the X‐ray beam aimed perpendicularly at the implants. The digital peri‐apical radiographs were utilized to assess potential crestal bone changes around each implant using an HP Scanjet 8300 Professional Image Scanner (Hewlett‐Packard, Palo Alto, CA, USA) and domain image processing software (ImageJ; US National Institutes of Health, Bethesda, MD, USA). The dimensions were calibrated using the known parameters of implant diameter and length. Vertical distances, measured in millimeters from the implant shoulder to the most apical initial point of first visible bone contact, were taken on the mesial and distal aspects of each implant (see Figure 2). Bone level measurements were conducted by an independent assessor who was blinded to the participant status throughout the clinical study. The blinded assessor was calibrated with the lead author, and repeat measures were conducted until the intraclass correlation coefficient reached > 95% before beginning the measurements.

### 2.8 PICF Sample Collection

The PICF samples were collected by the same operator at each follow‐up examination, from T1 to T9. To prevent salivary contamination, the selected crevicular sites were isolated using cotton rolls and dried with a gentle air spray. A standardized paper strip (Periopaper, ProFlow Inc., Amityville, NY, USA) was inserted 1 mm into the sulci at the three selected sites (mesial, buccal, and distal aspects of the provisional prosthesis or the abutments) and left in place for 30 s. The three paper strips were pooled and conditioned in separate RNase‐free EP tubes containing lysis solution. Subsequently, the eluate was centrifuged (for 5 min at 3000 g) to remove bacteria and cellular elements. Protein supernatants were collected and stored at −70°C for subsequent experiments.

### 2.9 ELISA Detection of the Cytokine Proteins OPG, RANKL, and CTS‐K in PICF

The commercial OPG, RANKL, and CTS‐K enzyme‐linked immunosorbent assay (ELISA) kits (from Wuhan Yunclone Technology Co. Ltd., China) were utilized to measure the levels of CTS‐K (ELISA kit number: P10180), RANKL (ELISA kit number: SEA855Hu), and OPG (ELISA kit number: SEA108Hu), following the manufacturers' instructions. The lowest detectable concentrations were set at 65 pg/mL for CTS‐K, 1 pg/mL for OPG, and 0.058 ng/mL for RANKL, respectively. The absorbance optical density (OD) value of each microplate containing the colored reaction liquid was measured at a wavelength of 450 nm using an enzyme‐labeled instrument. The corresponding concentrations were then calculated using the standard curve, following the manufacturer's instructions. The concentrations of these cytokines were expressed in nanograms per milliliter (ng/mL) for RANKL and in picograms per milliliter (pg/mL) for OPG and CTS‐K. The results were reported as total amounts (TA).

### 2.10 Real‐Time Detection of PRC for OPG mRNA and RANKL mRNA

The levels of OPG mRNA and RANKL mRNA were further assessed using real‐time fluorescence quantitative PCR due to their high sensitivity in ELISA. In the IR and CR groups, total RNA was extracted from PICF samples using the RNeasy Mini Kit (Qiagen, Hilden, Germany) following the manufacturer's protocol. The RNA concentrations were measured using the NanoDrop 2000/2000c ultra‐micro ultraviolet spectrophotometer (Thermo Fisher Scientific, USA). The samples and positive controls (standard curve) were processed according to the manufacturer's instructions, and the values for OPG RNA and RANKL RNA were calculated and reported as pg/mL.

A specified amount of OPG RNA or RANKL RNA was added to an enzyme‐free EP tube containing the RNA reverse transcription reaction system for cDNA synthesis, following the instructions provided with the Takara premix script RT kit from Takara Company, Japan. The primer sequences for OPG and RANKL, as well as their corresponding internal reference gene GAPDH, were designed and synthesized by the Sun Yat‐sen Medical College of Sun Yat‐sen University (refer to Table 1).

**Table 1:** Primers used in this study.

Gene	Primer sequence (5′‐3′)
*GAPDH*	The forward primer sequence TCACTGCCACCCAGAAGACT
	The reverse primer sequence TTCCCGTTCAGCTCAGGGAT
*OPG*	The forward primer sequence CACAAATTGCAGTGTCTTTGGTC
	The reverse primer sequence CTGCGTTTACTTTGGTGCCA
*RANKL*	The forward primer sequence CAACATATCGTTGGATCACAGCA
	The reverse primer sequence GACAGACTCACTTTATGGGAACC

For each target gene to be amplified, an equal volume of the PCR reaction system was added to separate EP tubes containing cDNA products, which were then reacted in the thermal cycler. The fluorescence signals of the amplification products were stored in the software of the real‐time fluorescence quantitative PCR instrument (LightCycler 480, Roche, USA) for analyzing the amplification curve. The relative gene expressions of OPG mRNA and RANKL mRNA in PICF were detected using the $2^{-∆∆\mathrm{CT}}$ method. The measurements were expressed as the expression levels from implants of the IR group being multiple times those from implants of the CR group.

### 2.11 Statistical Analyses

A descriptive analysis was conducted on continuous data, including cytokine levels, using measures such as the mean ± standard deviation (SD). The implant served as the statistical unit for the analysis. The heterogeneity of clinical parameters at both patient and implant levels within the IR and CR groups was assessed using the Student's *t*‐test or Fisher's exact test. The data for these cytokines and MBL were evaluated for homogeneity using the Shapiro–Wilk test. Subsequently, the expressions of cytokines and changes in MBL between the IR and CR groups were compared at scheduled time points using the independent *t*‐test. To analyze intergroup and intragroup differences in cytokine level variations during osseointegration and the 6‐month functional loading period, Repeated Measures ANOVA was applied if the data met normality criteria; otherwise, the Friedman test was utilized. The level of significance was set at 5%. The data analysis was performed using the statistical package SPSS version 15 (SPSS Inc., Chicago, IL, USA).

## 3 Results

### 3.1 Patients and Implants

A total of 56 individuals were consecutively recruited from a pool of patients requiring prosthodontic treatment for a single missing mandibular molar between January 2017 and December 2019. However, only forty‐nine patients (29 males and 20 females, aged 22–49 years; mean age of 36.4 ± 5.4 years) successfully completed the entire series of scheduled follow‐up visits and provided effective study data at both patient and implant levels. During the study period, three patients were withdrawn from the clinical trial due to intraoperative implant torque (IT) less than 30 Ncm, and four patients were removed because of substandard peri‐implant health indices. Of the remaining 49 eligible patients, 25 were assigned to the immediate loading (IR) group and 24 to the conventional loading (CR) group. Furthermore, 49 Zimmer TSV implants, with lengths of either 10 or 11.5 mm, depending on the available bone height, and a platform diameter of 4.1 mm, were placed in the healed molar sites of the mandibles. The implant survival rates for both the IR and CR groups at T9 (the study's end time) were 100%.

The remaining 49 qualified patients were well matched between the two groups in terms of sex, with 13 males and 12 females in the IR group, and 14 males and 10 females in the CR group. Additionally, the age distribution was comparable, with the IR group having a mean age of 37.4 ± 4.4 years and the CR group a mean age of 34.6 ± 5.2 years. The primary stability of all implants, which qualified for the study in both the IR and CR groups, ranged from 30 to 45 Ncm, as measured by the surgical motor of the implant machine. No statistically significant differences were observed between the IR and CR groups regarding sex, age, implant success rate, and implant dimensions (diameters and lengths) during the study period (*p* > 0.05, Table 2). The well‐matched characteristics of patients and dental implants between the two groups indicated that the intergroup heterogeneity of variable parameters at both patient and implant levels could not have caused an unfavorable impact on the study's clinical outcomes.

**Table 2:** Patients and implants characteristics in IR and CR group in the study.

	Immediate restoration group (IR, *n* = 25)	Conventional restoration group (CR, *n* = 24)	*p*
Age (years)^a^	37.4 ± 4.4	34.6 ± 5.2	0.07
Sex (male/female),^b^ *n*	13/12	14/10	0.65
The implant dimensions (diameter and length),^b^ *n*			0.78
4.1 × 11.5 mm, *n*	9	7	
4.1 × 10 mm, *n*	16	17	
Implant success^b^	100%	100%	1

*Note: n = *total of individuals.

^a^ Student *t* test (*p* < 0.05), mean ± standard deviation.

^b^ Fisher exact test (*p* < 0.05), %.

### 3.2 Radiographic Assessment of Alveolar Bone Loss Around the Evaluated Implants

Compared to the alveolar bone level at baseline (immediately after surgery), implants in the IR group exhibited a mean MBL of 0.64 ± 0.38 mm at the mesial aspect and 0.74 ± 0.41 mm at the distal aspect at T4, and 0.70 ± 0.37 mm at the mesial aspect and 0.68 ± 0.41 mm at the distal aspect at T9. In contrast, implants in the CR group demonstrated a mean MBL of 0.37 ± 0.07 mm at the mesial aspect and 0.35 ± 0.05 mm at the distal aspect at T4, and 0.65 ± 0.42 mm at the mesial aspect and 0.62 ± 0.43 mm at the distal aspect at T9. During the establishment of osseointegration, implants in the IR group exhibited mean MBL that was statistically significantly greater both mesially and distally compared to the CR group (*p* < 0.05), which may be associated with indirect mechanical stimuli from the nonfunctional loading of provisional prosthetic prostheses. Interestingly, following the delivery of the final prosthetic crown and full functional loading, the alveolar bone surrounding the implants in the IR group remained virtually unchanged, with no significant differences between the two groups in terms of mean mesial and distal MBL at T9 (*p* > 0.05). Additionally, throughout the entire study period, no implants in any group exhibited alveolar bone loss exceeding 1.5 mm.

### 3.3 Peri‐implant Health Index Assessment Throughout the Entire Study Period

**Figure 3:**
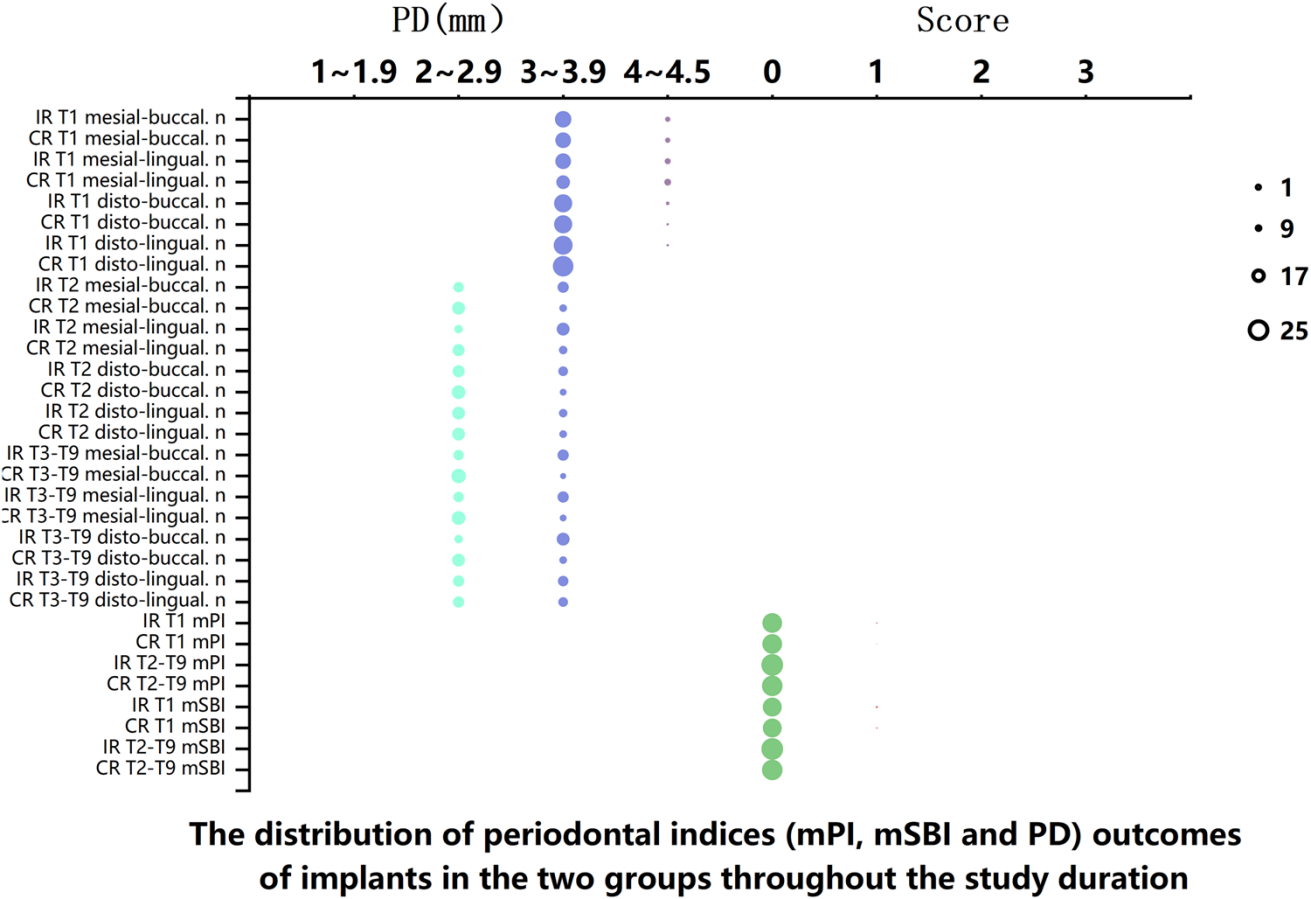
The distribution of periodontal indices (mPI, mSBI, and PD) among implants in the two groups over the study duration. Note: *n* represents the total number of individuals; T represents each follow‐up time point.

From T1 to T9, the peri‐implant healthy status was assessed using scores for mPI, mSBI, and PD. At T1, the majority of the soft tissue units around the implants did not bleed upon probing (Figure 3); however, in the IR group, 3 out of 25 implants (12%) and in the CR group, 2 out of 24 implants (8.3%) were scored as 1 by the mSBI criterion (Figure 3). From T2 to T9, no bleeding on probing occurred around the implants in both groups (Figure 3). Also at T1, 1 approximal surface of an implant in the IR group and 1 approximal surface of an implant in the CR group were found to carry obscure plaque, indicating that 8% (2/25) and 4.1% (1/24) of implants in the IR and CR groups, respectively, were scored 1 for mPI (Figure 3). From T2 to T9, a score of 0 for the mPI was recorded for all implants in both groups (Figure 3).

The mean PD values at the four sites around all implants in the two groups were reported to range from 2.0 to 4.5 mm throughout the study period (from T1 to T9; Figure 3). At T1, the mean PD values at the four sites around implants in both the IR and CR groups were between 3.0 and 4.5 mm. From T2 to T7, the mean PD values at the four sites around all implants in both groups varied between 2.5 and 4.0 mm.

Additionally, in both the IR and CR groups, the average probing depth (PD) of the implant at T1 was 1.0–1.5 mm deeper than at other follow‐up time points (from T2 to T9). In this study, the periodontal index results (Figure 3) and alveolar bone changes of less than 1.5 mm in both groups over the study period did not confirm the presence of peri‐implant mucositis and periodontitis, as judged by the consensus from the 2017 World Workshop on Peri‐Implant Diseases (Berglundh et al. 2018).

### 3.4 Detection of RANKL, OPG, CTS‐K, OPG mRNA, and RANKL mRNA in the PICF Sample

**Figure 4:**
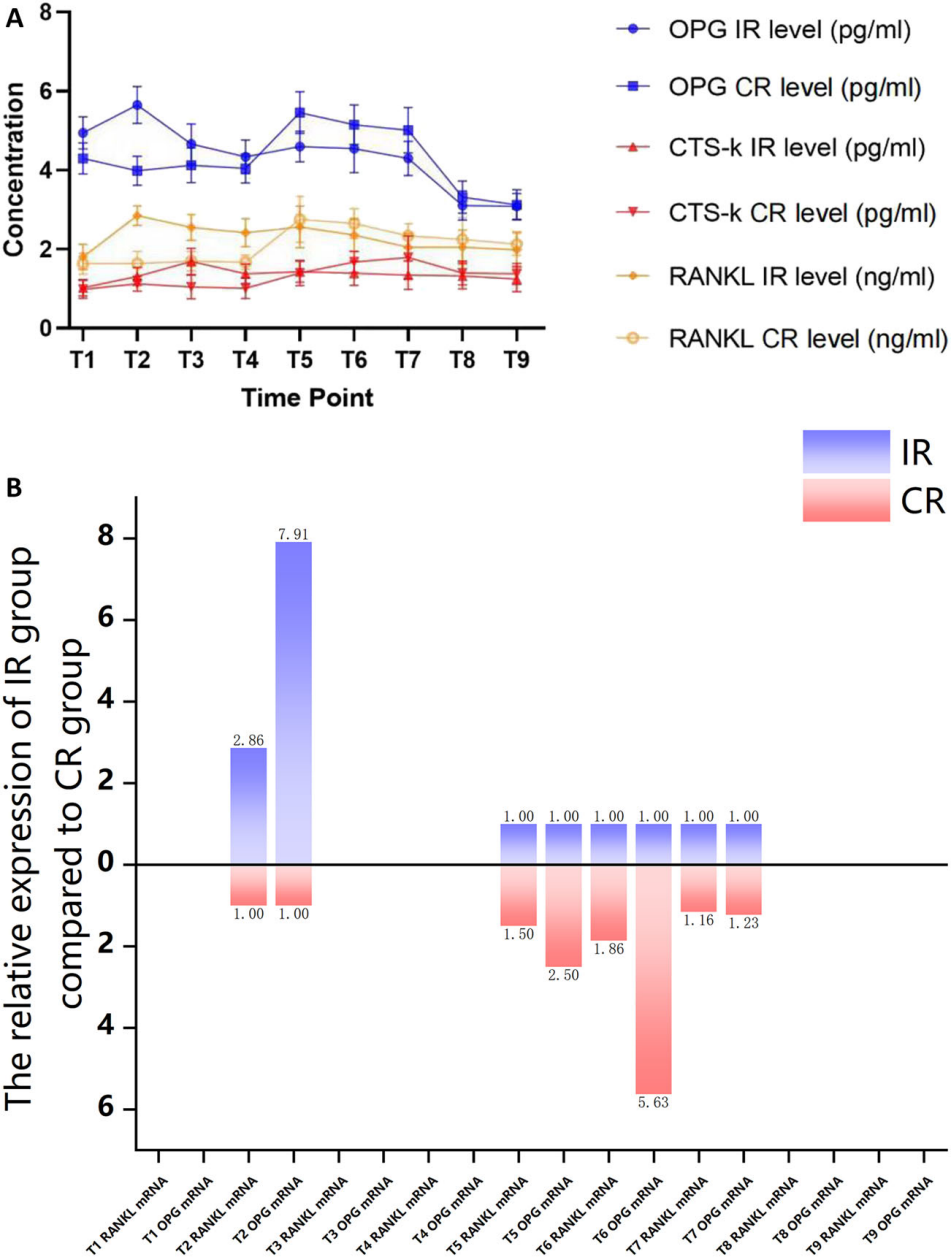
The levels of RANKL, OPG, and CTS‐K, as well as OPG mRNA and RANKL mRNA, at various scheduled time points in the IR and CR groups. (A) The level of RANKL, OPG, and CTS‐K at different scheduled timepoints in the IR group and the CR group. (B) The relative expression level of OPG mRNA and RANKL mRNA in the IR group against to the CR group.

During the osseointegration period (from T1 to T4), intragroup analyses indicated that RANKL, OPG, and CTS‐K levels showed significant fluctuations in the IR group and minor variations in the CR group (Figure 4A). Further intragroup analyses revealed that the highest release peaks of RANKL, OPG, and CTS‐K occurred at T2, T2, and T3, respectively, in the IR group (Figure 4A). These peaks were statistically significantly higher (*p* < 0.05) than those at other observation time points (Table 3).

**Table 3:** Intergroup analysis of the cytokines levels at different time points during nonsubmerged healing and functional loaded period, as well as intergroup analysis of the cytokines levels throughout the entire nonsubmerged healing and the entire functional loaded period.

Cytokines				RANKL	
Time (IR vs CR)	RANKL	OPG	CTS‐K	mRNA	OPG mRNA
Time points during nonsubmerged healing					
T1	*p* > **0.05** a	*p* < **0.05** a	*p* > **0.05** a	N	N
T2	***p*** < **0.05** a	***p*** < **0.05** a	*p* > **0.05** a	*p* < **0.05** a	*p* < **0.05** a
T3	*p* < **0.05** a	*p* > **0.05** a	*p* < **0.05** a	N	N
T4	*p* < **0.05** a	*p* > **0.05** a	*p* < **0.05** a	N	N
Time points during the loaded period					
T5	*p* > **0.05** a	*p* < **0.05** a	*p* > **0.05** a	*p* > **0.05** a	*p* > **0.05** a
T6	*p* > **0.05** a	*p* < **0.05** a	*p* > **0.05** a	*p* > **0.05** a	*p* < **0.05** a
T7	*p* > **0.05** a	*p* > **0.05** a	*p* < **0.05** a	*p* > **0.05** a	*p* > **0.05** a
T8	*p* > **0.05** a	*p* > **0.05** a	*p* > **0.05** a	N	N
T9	*p* > **0.05** a	*p* > **0.05** a	*p* > **0.05** a	N	N
Trend of cytokines change during nonsubmerged healing (from T1 to T4)	*p* < **0.05** b	*p* < **0.05** b	*p* < **0.05** b	N	N
Trend of cytokines change during loaded period (from T5 to T9)	*p* > **0.05** b	*p* > **0.05** b	*p* > **0.05** b	N	N

*Note: N* indicates no statistical analysis.

^a^ Student *t* test.

^b^ Repeated measures ANOVA.

The intergroup comparisons demonstrated that the IR group presented a statistically higher level (*p* < 0.05) from T2 to T4 regarding RANKL (Table 4), at T1 and T2 regarding OPG (Table 4), and at T3 and T4 regarding CTS‐K (Table 4). The further intergroup analyses revealed that the overall variation trend of these cytokines in the IR group during the osseointegration period was statistically different (*p* < 0.05) from that of the corresponding cytokines in the CR group (Table 4).

**Table 4:** Intragroup analysis of cytokines level change between different time points during nonsubmerged healing and functional loading period, as well as intragroup analysis of the trend of cytokines level change between the entire nonsubmerged healing and the entire functional loading period.

Cytokines	RANKL		OPG		CTS‐K	
Time	IR	CR	IR	CR	IR	CR
Between 4 time points during nonsubmerged healing (from T1 to T4)	*p* < 0.05	*p* > **0.05**	***p*** < **0.05**	*p* > **0.05**	***p*** < **0.05**	*p* > **0.05**
Between 5 time points during loaded period (from T5 to T9)	*p* > **0.05**	*p* < **0.05**	*p* > **0.05**	*p* < **0.05**	*p* > **0.05**	*p* < **0.05**
Trend of cytokines change during nonsubmerged healing period (from T1 to T4) vs. during loaded period (from T5 to T9)	*p* > **0.05**	*p* < **0.05**	*p* > **0.05**	*p* < **0.05**	*p* > **0.05**	*p* < **0.05**

*Note:* The statistical analysis results are all derived from Repeated Measures ANOVA.

After the final prosthesis delivery (from T5 to T9), the levels of RANKL, OPG, and CTS‐K showed a significant increase at T5 exclusively in the CR group, and then remained consistently elevated at T6 and T7 (Figure 4A). The intragroup analyses revealed that the levels of RANKL, OPG, and CTS‐K varied significantly from T5 to T9, with clear fluctuations in the CR group and minor variations in the IR group (Figure 4A). The highest peaks of RANKL, OPG, and CTS‐K release were observed at T5, T5, and T7, respectively, in the CR group (Figure 4A). Further intragroup analyses of the CR group revealed that the overall variation trends of RANKL, OPG, and CTS‐K during the osseointegration period (from T1 to T4) were statistically different (*p* < 0.05) from those of the corresponding cytokines during the 6 months of prosthetic loading (from T5 to T9) (Table 3). However, the overall variation trends of these cytokines observed in the CR group were not mirrored in the IR group (Figure 4A). Intergroup analyses indicated that during the 6 months of prosthetic loading (from T5 to T9), the overall variation trends of these three cytokines in the IR group were not statistically different (*p* > 0.05) from those of the corresponding cytokines in the CR group (Table 4).

Due to the weak ELISA levels of the three cytokines at certain observation time points in the present study, CTS‐K mRNA could not be further detected from T1 to T9 using real‐time fluorescence quantitative PCR; however, OPG mRNA and RANKL mRNA were only detectable at T2, T5, T6, and T7 by this method (Figure 4B). The relative expression levels of OPG mRNA and RANKL mRNA in the IR group compared to the CR group were measured and presented in Figure 4B. The expressions of OPG mRNA and RANKL mRNA were significantly higher (*p* < 0.05) at T2 in the IR group compared to the CR group, which corresponded to the levels of OPG and RANKL being significantly higher at T2 (*p* < 0.05) in the IR group than in the CR group (Table 4). Conversely, the expressions of OPG mRNA and RANKL mRNA were higher at T5, T6, and T7 in the CR group compared to the IR group, which mutually verified that the levels of OPG and RANKL during T5 to T7 were higher in the CR group than in the IR group. However, only the intergroup differences in the expressions of OPG mRNA at T6 were statistically significant (*p* < 0.05) (Table 4).

## 4 Discussion

In the current study, all evaluated implants were placed in the molar region of the mandible, where high‐quality bone is typically found, facilitating the attainment of proper initial implant stability. Clinically, the proportion of subjects requiring single molar implant restoration in the mandible is the highest among patients missing a single tooth, which holds significant representative value. Furthermore, recruiting the required sample size for studies involving this population can be relatively easy and can be achieved within a short time frame. All evaluated implants in this study were placed in the same location to avoid sample selection bias due to varying jawbone regions. Moreover, the statistical analysis revealed no significant intergroup heterogeneity of variables at both patient and implant levels, suggesting that this heterogeneity may not negatively impact the study's explanatory power.

The osseointegration process begins with bone damage resulting from surgical injuries during the preparation of the implant site. This initially triggers the activation of osteoclasts, which remove the damaged bone (Giannelli et al. 2009). New bone forms on the implant's surface as osteogenic cells differentiate, migrate, and continue to build a bridge between the implant and the bone (Yorioka et al. 2024). At this stage, osteoclasts specialize in bone resorption, breaking down old or damaged bone around the newly inserted implant, which allows room for osteoblasts to deposit new bone. This ensures the maintenance of bone quality and facilitates adaptation at the bone‐implant interface (Han et al. 2014). Osteoblasts and osteoclasts both play a central role during the formation or remodeling phase of osseointegration.

In the immediate restoration period, bone tissue physiologically undergoes remodeling, which is the result of a dynamic balance between bone formation by osteoblasts and resorption by osteoclasts. Furthermore, bone formation and resorption may respond to dynamic indirect mechanical occlusal forces from the immediate restoration; the identification of molecular sensors, transducers, and signaling pathways involved in these processes is of utmost importance. Numerous cellular proteins sense, transduce, and integrate environmental signals, playing roles in the various phases of bone remodeling and being susceptible to mechanical stimuli. The OPG/RANKL/RANK system is considered the main signaling pathway accountable for bone metabolism and governing bone remodeling (Houchen et al. 2023; Hu et al. 2019; Kapasa et al. 2017).

The process of osseointegration is influenced by the release of specific cytokines, such as RANKL, OPG, and CTSK. Within a few hours following the placement of an implant, inflammatory cells migrate to the implant's surface, initiating the inflammatory response (Tumedei et al. 2020; Weivoda and Bradley 2023; Tater and Diajil 2023). The inflammatory response gradually triggers the release of RANKL from osteoblasts in the alveolar bone; this RANKL subsequently promotes the differentiation of osteoclast precursor cells into osteoclasts, initiating bone resorption to eliminate damaged and necrotic tissue around the implant and preventing osteoclast apoptosis. Concurrently, osteoblasts secrete OPG, which acts as a decoy receptor for RANKL, binding to it and inhibiting the differentiation and maturation of osteoclast precursor cells, thereby gradually achieving a balance between bone formation and remodeling resorption (Hu et al. 2019; Han et al. 2014). RANKL and OPG participate in the regulation of bone remodeling through the OPG/RANKL signaling pathway (Liu et al. 2020). Additionally, CTS‐K is a cysteine protease primarily secreted by osteoclasts (Martin and Sims 2015; Shankar et al. 2024)and regulated by RANKL (Kapasa et al. 2017). CTS‐K participates in mediating the dynamic balance of bone remodeling by facilitating the degradation of the bone's organic matrix (Boraschi‐Diaz et al. 2018). Therefore, the dynamic changes in OPG, RANKL, and CTS‐K can, to some extent, reflect the state of peri‐implant bone metabolism (Tian and Fan 2015).

During the nonsubmerged healing (osseointegration) period of this study, levels of RANKL, OPG, and CTS‐K were detected at four observation time points in both IR and CR groups. The overall trends of these three cytokine expression levels were statistically higher in the IR group compared to the CR group, suggesting that mechanical occlusal forces from immediate restoration may increase the release of these cytokines, thereby accelerating peri‐implant bone metabolism and promoting the maturation of the implant‐bone interface by early enlivening bone remodeling.

Further intergroup analysis revealed statistically significant differences in RANKL and OPG expression levels at T2, and in CTS‐K expression levels at T3 and T4 during the osseointegration period. These findings suggest that occlusal stimulation from immediate restoration may promote bone formation and remodeling by stimulating bone metabolism at critical stages of bone healing. This is attributed to T2 representing the period of active bone formation, and T3 and T4 representing the period of gradually active bone remodeling. Furthermore, intragroup analysis within the IR group revealed that the highest levels of RANKL and OPG at T2, and CTS‐K at T3, were statistically significantly higher than the expression levels of the corresponding cytokines at other time points within the same group. In contrast, within the CR group, there were no significant differences in the expression levels of the three cytokines at the four observation time points. These results once again validated that the variation in the three cytokines at the four time points within both groups was likely ascribed to the occlusion factor. Taken together, the higher expression levels of cytokines in the IR group during the osseointegration period may indicate higher tissue remodeling activity.

In this study, the overall trends of RANKL, OPG, and CTS‐K release showed a statistically significant increase (*p* < 0.05) within the first 6 months following the final restoration in the CR group, compared to the levels during the osseointegration period. However, this was not observed in the IR group. The results indicated that in the IR group, after 3 months of indirect physiological functional occlusal stimulation during the osseointegration period, the peri‐implant bone tissue gradually adapted to this stimulation intensity and was no longer able to release these three cytokines at high levels. These findings suggest that the effect of mechanical stimulation on the release of these three cytokines is effective only during the early stages following occlusal mechanical stimulation intervention, and this effect diminishes as loading progresses.

Over the 6‐month observation period following permanent restoration, the CR group exhibited varying degrees of increased expression levels of OPG, RANKL, and CTS‐K at the five time points, reaffirming that mechanical stimulation can enhance cytokine release levels. During this period, the levels of OPG at T5 and T6, and CTS‐K at T7 were significantly higher in the CR group compared to the IR group at the corresponding time points; however, there was no statistically significant difference in the trend of these cytokine expression levels between the two groups. These results suggest that the effect of mechanical stimulation on the cytokine release of bone tissue is time‐dependent, occurring only during the initial phase after mechanical stimulation intervention.

Since RANKL, OPG, and CTSK play distinct roles in bone formation and remodeling, their expression levels are adjusted at various stages to regulate the rate of bone tissue formation and remodeling. This study indicates that elevated release levels of RANKL and OPG predominantly occur during the early phase of nonsubmerged healing, whereas increased release levels of CTSK are observed during the later phase of nonsubmerged healing and the initial phase following permanent restoration. These dynamic alterations in cytokine expression levels correspond with the physiological changes in bone metabolism that take place during the establishment and remodeling of the implant‐bone interface. Based on these research findings, it is speculated that mechanical stimulation enhances bone metabolism by modulating cytokine release, thereby influencing the establishment and maturation of the implant‐bone interface.

Additionally, the detection of cytokines at the gene level revealed that the expression levels of OPG mRNA and RANKL mRNA at T2 were significantly higher in the IR group compared to the CR group. Conversely, at T5 and T6, the expression levels of OPG mRNA and RANKL mRNA were significantly higher in the CR group than in the IR group. These findings may offer a plausible explanation for the impact of mechanical stimuli on the implant‐bone interface, which primarily functions during the initial phase following mechanical stimulation intervention. By regulating cytokine release, mechanical stimuli can influence bone metabolism.

In this study, it was intriguing to observe that the MBL was significantly lower—less than 1.0 mm—mesially and distally only during the nonsubmerged healing (osseointegration) phase in the IR group compared to the conventional loading CR group. This suggests that the physiological metabolic activity of the surrounding bone tissue during the formation of the implant‐bone interface is highly sensitive to the occlusal forces applied in the IR group. The bone metabolic balance is negatively impacted by improper mechanical stimulation from occlusion, leading to microfractures in the peri‐implant bone tissue. This, in turn, results in limited physiological bone resorption while promoting the establishment of the implant‐bone interface.

Regular maintenance of implants is crucial to monitor the peri‐implant situation throughout the entire research process. When sampling gingival crevicular fluid around the implant, it is important to avoid contamination by microorganisms in the fluid to ensure the accuracy of cytokine detection. Throughout the study, oral care supervision was consistently enhanced, resulting in no significant anomalies in PD values, mPI scores, or mSBI scores among all eligible subjects. This indicates that healthy peri‐implant conditions had no adverse effects on cytokine sampling in PICF and the marginal bone around implants during the study period. Moreover, the clinical periodontal parameter outcomes and minor changes in peri‐implant alveolar bone in the two groups confirmed no signs of peri‐implant mucositis and periodontitis during the entire study period, which accordingly supports the effectiveness of the explanation of the osseointegration establishment of the immediately restored implant using results from the three cytokines.

The implant established an initial bone‐implant interface following 3 months of bone healing in the absence of load. This newly formed bone interface requires gradual maturation through bone remodeling under physiological masticatory mechanical stimulation. Repeated mechanical occlusal stimuli are crucial for the successful formation of the implant‐bone interface. The study's results regarding RANKL, OPG, CTSK, RANKLmRNA, and OPGmRNA expression changes indicate that indirect occlusion stimulation from immediate restoration may trigger cellular stress responses and mechanosensory signals, leading to the active release of RANKL, OPG, and CTSK. This likely prompts the maturation of the implant‐bone interface by regulating bone metabolism during the osseointegration phase. Therefore, based on previous histological findings on the osseointegration of implants undergoing immediate restoration, it can be inferred that the rapid maturation of the implant‐bone interface is closely related to physiological occlusal stimulation, particularly to the active release of cytokines associated with bone metabolism under occlusal mechanical stimulation. Furthermore, the results of this study can serve as evidence for molecular biology research supporting the view that physiological mechanical stimulation during immediate restoration aids in accelerating the establishment and maturation of the implant‐bone interface.

The limitations of this study included inconsistent patient compliance with dietary control and smoking among the subjects during the 3‐month osseointegration period. Furthermore, patients were not scheduled for sample collection at the same time of day to avoid cyclical variations affecting crevicular fluid volume, potentially introducing selection bias and confounding factors. Another drawback was the failure to utilize a radiographic stent to standardize the x‐rays and assess three‐dimensional changes in peri‐implant bone levels through two‐dimensional radiographs. Additionally, recruiting a sufficient number of patients who met the strict inclusion criteria, such as oral hygiene standards, and maintaining patient homogeneity within the short study period was challenging, leading to a small sample size. However, studies with a larger number of patients are required. Moreover, the study lacked detection of CTS‐K mRNA expression levels, which partly diminished the effectiveness of the research data analysis. Consequently, the outcomes of this study cannot be generalized beyond its specific domain.

## 5 Conclussions

The results of the current study indicate that immediate restoration occlusal mechanical stimulation may positively affect the establishment and maturation of osseointegration by regulating the release of RANKL, OPG, and CTSK through mechanical‐sensitive cellular events.

## Author Contributions

Xiaowen HU contributed to conception and design, analysis, and interpretation of this study, and critically revised the manuscript. yijie Fan contributed to the original data of the trials, analysis and interpretation of this study, and drafted the manuscript. Xuexia Chen contributed to the original data of the trials, analysis, and interpretation of this study. All authors gave their final approval and agreed to be held accountable for all aspects of the work, ensuring integrity and accuracy.

## Ethics Statement

The research protocol was approved by the Human Ethics Review Committee of the Stomatological Hospital, Sun Yat‐sen University of Guanghua Oral Medical College (KQEC‐2021‐45‐02).

## Conflicts of Interest

The authors declare no conflicts of interest.

## Data Availability

The data that support the findings of this study are available from the corresponding author upon reasonable request. Supporting information, such as raw data, is available from the corresponding author upon reasonable request.

## References

[cre270193-bib-0001] Andrade, C. A. S. , J. L. C. Paz , G. S. de Melo , N. Mahrouseh , A. L. Januário , and L. R. Capeletti . 2022. “Survival Rate and Peri‐Implant Evaluation of Immediately Loaded Dental Implants in Individuals With Type 2 Diabetes Mellitus: A Systematic Review and Meta‐Analysis.” Clinical Oral Investigations 26, no. 2: 1797–1810. 34586502 10.1007/s00784-021-04154-6 PMC8479496

[cre270193-bib-0002] Attard, N. J. , and G. A. Zarb . 2005. “Immediate and Early Implant Loading Protocols: A Literature Review of Clinical Studies.” Journal of Prosthetic Dentistry 94, no. 3: 242–258. 16126077 10.1016/j.prosdent.2005.04.015

[cre270193-bib-0003] Balu, P. , A. K. Balakrishna Pillai , V. Mariappan , and S. Ramalingam . 2024. “Cytokine Levels in Gingival Tissues as an Indicator to Understand Periodontal Disease Severity.” Current Research in Immunology 5: 100080. 39026560 10.1016/j.crimmu.2024.100080 PMC11254528

[cre270193-bib-0004] Berglundh, T. , G. Armitage , M. G. Araujo , J. Wennström , and G. Romanos . 2018. “Peri‐Implant Diseases and Conditions: Consensus Report of Workgroup 4 of the 2017 World Workshop on the Classification of Periodontal and Peri‐Implant Diseases and Conditions.” Journal of Periodontology 89, no. Suppl 1: S313–S318. 29926955 10.1002/JPER.17-0739

[cre270193-bib-0005] Bonato, R. S. , G. V. O. Fernandes , M. D. Calasans‐Maia , et al. 2022. “The Influence of rhBMP‐7 Associated With Nanometric Hydroxyapatite Coatings Titanium Implant on the Osseointegration: A Pre‐Clinical Study.” Polymers 14, no. 19: 4030. 10.3390/polym14194030. 36235978 PMC9570843

[cre270193-bib-0006] Boraschi‐Diaz, I. , J. S. Mort , D. Brömme , Y. A. Senis , A. Mazharian , and S. V. Komarova . 2018. “Collagen Type I Degradation Fragments Act Through the Collagen Receptor LAIR‐1 to Provide a Negative Feedback for Osteoclast Formation.” Bone 117, no. 1: 23–30. 30217615 10.1016/j.bone.2018.09.006

[cre270193-bib-0007] Charifker Ribeiro Martins, S. , M. C. Marques , M. G. Vidal , et al. 2024. “Is the Facial Bone Wall Critical to Achieving Esthetic Outcomes in Immediate Implant Placement With Immediate Restoration? A Systematic Review.” Advances in Clinical and Experimental Medicine 33, no. 9: 979–997. 38180330 10.17219/acem/173573

[cre270193-bib-0008] Cordioli, G. , Z. Majzoub , A. Piattelli , G. Iezzi , and J. A. Shibli . 2000. “Removal Torque and Histomorphometric Investigation of 4 Different Titanium Surfaces: An Experimental Study in the Rabbit Tibia.” International Journal of Oral & Maxillofacial Implants 15, no. 5: 668–674. 11055134

[cre270193-bib-0009] Duarte, P. M. , C. R. Serrão , T. S. Miranda , et al. 2016. “Could Cytokine Levels in the Peri‐Implant Crevicular Fluid Be Used to Distinguish Between Healthy Implants and Implants With Peri‐Implantitis? A Systematic Review.” Journal of Periodontal Research 51, no. 6: 689–698. 26774043 10.1111/jre.12354

[cre270193-bib-0010] Fernandes, G. , B. Costa , H. Trindade , R. Castilho , and J. Fernandes . 2022. “Comparative Analysis Between Extra‐Short Implants (≤ 6 mm) and 6 mm‐Longer Implants: A Meta‐Analysis of Randomized Controlled Trial.” Australian Dental Journal 67, no. 3: 194–211. 35094419 10.1111/adj.12900

[cre270193-bib-0011] Fernandes, G. , N. Ferreira , A. Heboyan , L. Nassani , R. Pereira , and J. Fernandes . 2023. “Clinical Assessment of Short (> 6 mm and ≤ 8.5 mm) Implants in Posterior Sites With An Average Follow‐Up of 74 Months: A Retrospective Study.” International Journal of Oral & Maxillofacial Implants 38, no. 5: 915–926. 37847833 10.11607/jomi.10197

[cre270193-bib-0012] Ferreira Balan, V. , M. Ferri , E. Pires Godoy , et al. 2024. “Controlled Lateral Pressure on Cortical Bone Using Blade‐Equipped Implants: An Experimental Study in Rabbits.” Bioengineering 11, no. 8: 835. 10.3390. 39199793 10.3390/bioengineering11080835 PMC11352121

[cre270193-bib-0013] Gehrke, S. A. , B. A. Dedavid , and G. V. de Oliveira Fernandes . 2021. “A New Design of a Multifunctional Abutment to Morse Taper Implant Connection: Experimental Mechanical Analysis.” Journal of the Mechanical Behavior of Biomedical Materials 116: 104347. 33513461 10.1016/j.jmbbm.2021.104347

[cre270193-bib-0014] Gheisary, Z. , R. Mahmood , A. Harri Shivanantham , et al. 2022. “The Clinical, Microbiological, and Immunological Effects of Probiotic Supplementation on Prevention and Treatment of Periodontal Diseases: A Systematic Review and Meta‐Analysis.” Nutrients 14, no. 5: 1036. 10.3390. 35268009 10.3390/nu14051036 PMC8912513

[cre270193-bib-0015] Giannelli, M. , D. Bani , A. Tani , et al. 2009. “In Vitro Evaluation of the Effects of Low‐Intensity Nd:YAG Laser Irradiation on the Inflammatory Reaction Elicited by Bacterial Lipopolysaccharide Adherent to Titanium Dental Implants.” Journal of Periodontology 80, no. 9: 977–984. 19485829 10.1902/jop.2009.080648

[cre270193-bib-0016] Hadzik, J. , K. Jurczyszyn , T. Gębarowski , et al. 2023. “An Experimental Anodized and Low‐Pressure Oxygen Plasma‐Treated Titanium Dental Implant Surface‐Preliminary Report.” International Journal of Molecular Sciences 24, no. 4: 3603. 10.3390. 36835015 10.3390/ijms24043603 PMC9958761

[cre270193-bib-0017] Han, J. , J. Hou , G. Zhou , C. Wang , and Y. Fan . 2014. “A Histological and Biomechanical Study of Bone Stress and Bone Remodeling Around Immediately Loaded Implants.” Science China Life Sciences 57, no. 5: 618–626. 24824585 10.1007/s11427-014-4657-7

[cre270193-bib-0018] Hong, J. M. , U. G. Kim , and I. S. L. Yeo . 2022. “Comparison of Three‐Dimensional Digital Analyses and Two‐Dimensional Histomorphometric Analyses of the Bone‐Implant Interface.” PLoS One 17, no. 10: e0276269. 10.1371. 36240217 10.1371/journal.pone.0276269 PMC9565376

[cre270193-bib-0019] Houchen, C. J. , B. Castro , P. Hahn Leat , N. Mohammad , F. Hall‐Glenn , and E. E. Bumann . 2023. “Treatment With An Inhibitor of Matrix Metalloproteinase 9 or Cathepsin K Lengthens Embryonic Lower Jaw Bone.” Orthodontics & Craniofacial Research 26, no. 3: 500–509. 36680416 10.1111/ocr.12635 PMC11508777

[cre270193-bib-0020] Hu, Z. , D. Wu , Y. Zhao , S. Chen , and Y. Li . 2019. “Inflammatory Cytokine Profiles in the Crevicular Fluid Around Clinically Healthy Dental Implants Compared to the Healthy Contralateral Side During the Early Stages of Implant Function.” Archives of Oral Biology 108: 104509. 31494437 10.1016/j.archoralbio.2019.104509

[cre270193-bib-0021] Iezzi, G. , S. Orlandi , G. Pecora , and A. Piattelli . 2009. “Histologic and Histomorphometric Evaluation of the Bone Response Around a Hydroxyapatite‐Coated Implant Retrieved After 15 Years.” International Journal of Periodontics and Restorative Dentistry 29, no. 2: 99–105. 19244887

[cre270193-bib-0022] Iezzi, G. , G. Pecora , A. Scarano , A. Piattelli , and G. Papadopulos . 2009. “Immediately Loaded Screw Implant Retrieved After a 12‐year Loading Period: A Histologic and Histomorphometric Case Report.” Journal of Osseointegration 1, no. 1: 54–59.

[cre270193-bib-0023] Kanayama, M. , M. Ferri , F. M. M. Guzon , et al. 2024. “Influence on Marginal Bone Levels at Implants Equipped With Blades Aiming to Control the Lateral Pressure on the Cortical Bone. An Experimental Study in Dogs.” Oral and Maxillofacial Surgery 28, no. 3: 1139–1149. 38429433 10.1007/s10006-024-01228-z

[cre270193-bib-0024] Kapasa, E. R. , P. V. Giannoudis , and X. Jia . 2017. “The Effect of RANKL/OPG Balance on Reducing Implant Complications.” Journal of Functional Biomaterials 8, no. 1: 1–10. 28937598 10.3390/jfb8040042 PMC5748549

[cre270193-bib-0025] Kozakiewicz, M. , and T. Wach . 2022. “Exploring the Importance of Corticalization Occurring in Alveolar Bone Surrounding a Dental Implant.” Journal of Clinical Medicine 11, no. 23: 7189. 10.3390. 36498764 10.3390/jcm11237189 PMC9738071

[cre270193-bib-0026] Kuwano, K. , L. Canullo , D. Botticelli , et al. 2025. “Ablative and Expansive Protocols for Bone Osteotomy in Rabbits.” Dentistry Journal 13, no. 3: 118. 10.3390. 40136746 10.3390/dj13030118 PMC11941037

[cre270193-bib-0027] Liu, Y. , Q. Liu , Z. Li , et al. 2020. “Long Non‐Coding RNA and mRNA Expression Profiles in Peri‐Implantitis vs Periodontitis.” Journal of Periodontal Research 55, no. 3: 342–353. 31853997 10.1111/jre.12718

[cre270193-bib-0028] Lozada, J. L. , N. Tsukamoto , A. Farnos , J. Kan , and K. Rungcharassaeng . 2000. “Scientific Rationale for the Surgical and Prosthodontic Protocol for Immediately Loaded Root Form Implants in the Completely Edentulous Patient.” Journal of Oral Implantology 26, no. 1: 51–59. 11831303 10.1563/1548-1336(2000)026<0051:SRFTSA>2.3.CO;2

[cre270193-bib-0029] Mangano, F. G. , G. Iezzi , J. A. Shibli , et al. 2017. “Early Bone Formation Around Immediately Loaded Implants With Nanostructured Calcium‐Incorporated and Machined Surface: A Randomized, Controlled Histologic and Histomorphometric Study in the Human Posterior Maxilla.” Clinical Oral Investigations 21, no. 7: 2603–2611. 28154996 10.1007/s00784-017-2061-y

[cre270193-bib-0030] Martin, T. J. , and N. A. Sims . 2015. “RANKL/OPG: Critical Role in Bone Physiology.” Reviews in Endocrine and Metabolic Disorders 16, no. 2: 131–139. 25557611 10.1007/s11154-014-9308-6

[cre270193-bib-0031] Mombelli, A. , and N. P. Lang . 1994. “Clinical Parameters for the Evaluation of Dental Implants.” Periodontology 2000 4, no. 1: 81–86. 9673196 10.1111/j.1600-0757.1994.tb00008.x

[cre270193-bib-0032] Moraschini, V. , and E. Porto Barboza . 2016. “Immediate Versus Conventional Loaded Single Implants in the Posterior Mandible: A Meta‐Analysis of Randomized Controlled Trials.” International Journal of Oral and Maxillofacial Surgery 45, no. 1: 85–92. 26259980 10.1016/j.ijom.2015.07.014

[cre270193-bib-0033] Morena, D. , B. Leitão‐Almeida , M. Pereira , et al. 2024. “Comparative Clinical Behavior of Zirconia Versus Titanium Dental Implants: A Systematic Review and Meta‐Analysis of Randomized Controlled Trials.” Journal of Clinical Medicine 13, no. 15: 4488. 10.3390. 39124755 10.3390/jcm13154488 PMC11313197

[cre270193-bib-0034] Mukherjee, S. , S. Rodrigues , M. M , et al. 2022. “Biomechanical Stress Analysis of Platform Switch Implants of Varying Diameters on Different Densities of Bone.” International Journal of Dentistry 2022: 5972259. 10.1155/2022/5972259. 35251181 PMC8894074

[cre270193-bib-0035] de Oliveira Fernandes, G. , N. Santos , M. de Sousa , and J. Fernandes . 2022. “Liquid Platelet‐Rich Fibrin Coating Implant Surface to Enhance Osseointegration: A Double‐Blinded, Randomized Split‐Mouth Trial With 1‐Year Follow‐Up.” International Journal of Oral & Maxillofacial Implants 37, no. 1: 159–170. 35235635 10.11607/jomi.9107

[cre270193-bib-0036] Remísio, M. , T. Borges , F. Castro , S. Gehrke , J. Fernandes , and G. Fernandes . 2023. “Histologic Osseointegration Level Comparing Titanium and Zirconia Dental Implants: Meta‐Analysis of Preclinical Studies.” International Journal of Oral & Maxillofacial Implants 38, no. 4: 667–680. 37669522 10.11607/jomi.10142

[cre270193-bib-0037] Rondone, E. M. , B. Leitão‐Almeida , M. S. Pereira , G. V. O. Fernandes , and T. Borges . 2024. “The Use of Tissue Grafts Associated With Immediate Implant Placement to Achieve Better Peri‐Implant Stability and Efficacy: A Systematic Review and Meta‐Analysis.” Journal of Clinical Medicine 13, no. 3: 821. 38337515 10.3390/jcm13030821 PMC10856075

[cre270193-bib-0038] Shankar, R. , A. Saha , R. S. Dhull , R. Shroff , A. Nangia , and S. Sharma . 2024. “Activin A: A Marker of Mineral Bone Disorder in Children With Chronic Kidney Disease?” Pediatric Nephrology 39, no. 9: 2773–2777. 10.1007. 38744714 10.1007/s00467-024-06400-x

[cre270193-bib-0039] Di Stefano, D. , G. Iezzi , A. Scarano , A. Piattelli , and M. Piattelli . 2006. “Immediately Loaded Blade Implant Retrieved From Man After a 20 Years Loading Period: A Histological and Histomorphometrical Case Report.” Journal of Oral Implantology 32, no. 3: 171–176. 17009561 10.1563/765.1

[cre270193-bib-0040] Tater, J. , and A. R. Diajil . 2023. “Immunohistochemical Analysis of Osteoclastic and Osteoblastic Activity in Ossifying Fibroma and Juvenile Ossifying Fibroma: A Comparative Study.” Journal of Medicine and Life 16, no. 9: 1369–1374. 38107708 10.25122/jml-2023-0126 PMC10719798

[cre270193-bib-0041] Tian, H. , and Y. B. Fan . 2015. “Structure, Mechanism of Action and Role of OPG, RANK, and RANKL in Bone Diseases.” Progress in Modern Biomedicine 10, no. 12: 3963–3966.

[cre270193-bib-0042] Tumedei, M. , A. Piattelli , M. Degidi , C. Mangano , and G. Iezzi . 2020. “A Narrative Review of the Histological and Histomorphometrical Evaluation of the Peri‐Implant Bone in Loaded and Unloaded Dental Implants. A 30‐Year Experience (1988–2018).” International Journal of Environmental Research and Public Health 17, no. 6: 2088. 10.3390. 32245226 10.3390/ijerph17062088 PMC7143607

[cre270193-bib-0043] Weivoda, M. M. , and E. W. Bradley . 2023. “Macrophages and Bone Remodeling.” Journal of Bone and Mineral Research 38, no. 3: 359–369. 36651575 10.1002/jbmr.4773 PMC10023335

[cre270193-bib-0044] Yamagata, K. , Y. Oga , S. Kwon , A. Maeda‐Iino , T. Ishikawa , and S. Miyawaki . 2023. “A Novel Auxiliary Device Enhances Miniscrew Stability under Immediate Heavy Loading Simulating Orthopedic Treatment.” Angle Orthodontist 93, no. 1: 71–78. 36126677 10.2319/022222-163.1 PMC9797141

[cre270193-bib-0045] Yorioka, H. , Y. Otsu , R. Suzuki , et al. 2024. “The Influence of Immediate Occlusal Loading on Micro/Nano‐Structure of Peri‐Implant Jaw Bone in Rats.” International Journal of Implant Dentistry 10, no. 1: 24. 10.1186. 38722448 10.1186/s40729-024-00538-x PMC11082111

